# A comprehensive multivariate approach for GxE interaction analysis in early maturing rice varieties

**DOI:** 10.3389/fpls.2024.1462981

**Published:** 2024-10-01

**Authors:** Muhammad Fuad Anshori, Yunus Musa, Muh Farid, Muh Jayadi, Rusnadi Padjung, Kaimuddin Kaimuddin, Yi Cheng Huang, Madonna Casimero, Iris Bogayong, Willy Bayuardi Suwarno, Hasil Sembiring, Bambang Sapta Purwoko, Amin Nur, Wahyuni Wahyuni, Daniel O. Wasonga, Mahmoud F. Seleiman

**Affiliations:** ^1^ Department of Agronomy, Faculty of Agriculture, Hasanuddin University, Makassar, Indonesia; ^2^ Department of Soil Science, Faculty of Agriculture, Hasanuddin University, Makassar, Indonesia; ^3^ Taiwan International Cooperation and Development Fund (TaiwanICDF), Taipei, Taiwan; ^4^ International Rice Research Institute, University of the Philippines Los Baños, Los Baños, Philippines; ^5^ Department of Agronomy and Horticulture, Faculty of Agriculture, IPB University, Bogor, Indonesia; ^6^ Research Center for Food Crops, Research Organization for Agriculture and Food, National Research and Innovation Agency, Cibinong, Indonesia; ^7^ Indonesian Cereal Testing Instrument Standard Institute, Maros, South Sulawesi, Indonesia; ^8^ Food Crops, Horticulture, Plantation and Food Security Office of Soppeng, Soppeng, Indonesia; ^9^ Department of Crop Sciences, University of Illinois Urbana-Champaign, Urbana, IL, United States; ^10^ Plant Production Department, College of Food and Agriculture Sciences, King Saud University, Riyadh, Saudi Arabia

**Keywords:** BLUP, early-maturity rice, GxE analysis, index regression, oryza sativa

## Abstract

The genotype evaluation process requires analysis of GxE interactions to ascertain the responsiveness of a genotype to various environments, including the development of early maturing rice. However, the concept of interaction is relatively specific to grain yield. In contrast, grain yield is highly polygenic, so assessment should be carried out with multivariate approaches. Therefore, multivariate assessment in evaluating GxE interactions should be developed, especially for early maturing rice genotypes. The study aimed to develop a comprehensive multivariate approach to improve the comprehensiveness and responsiveness of GxE interaction analysis. The study was conducted in Bone and Soppeng districts, South Sulawesi, Indonesia, in two seasons. The study used a randomized complete block design, where replications were nested across two seasons and locations. Two check varieties and five early maturing varieties were replicated three times in each environment. Based on this study, a new approach to GxE interaction analysis based on multiple regression index analysis, BLUP analysis, factor analysis, and path analysis was considered adequate, especially for evaluating early maturing rice. This approach combined days to harvest, biological yield, and grain yield in multiple linear regression with weighting based on the combination of all analyses. The effectiveness of the GxE interaction assessment was reflected by high coefficient of determination (R^2^) and gradient (b) values above 0.8 and 0.9, respectively. Inpari 13 (R^2 = ^0.9; b=1.05), Cakrabuana (R^2 = ^0.98; b=0.99), and Padjajaran (R^2 = ^0.95; b=1.07) also have good grain yield with days to harvesting consideration, namely 7.83 ton ha^-1^, 98.12 days; 7.37 ton ha^-1^, 95.52 days; and 7.29 ton ha^-1^, 97.23 days, respectively. Therefore, this index approach can be recommended in GxE interaction analysis to evaluate early maturing rice genotypes. Furthermore, Inpari 13, Cakrabuana, and Padjajaran are recommended as adaptive early maturing varieties.

## 1 Introduction

Rice is a major food crop that has always been prioritized for development. This crop has the advantage of grain content rich in carbohydrates and several other components, such as vitamins, antioxidants, and minerals (Fukagawa and Ziska, 2019; Sen et al., 2020). These ingredients work synergistically to support the availability of energy and health for humans, so this crop is often consumed as the primary source of carbohydrates for most of the world’s population, including Indonesia. Indonesia belongs to the world’s top five rice production countries, after China, India, and Bangladesh (Yuan et al., 2022; Bin Rahman and Zhang, 2023). According to Statistic Indonesia (2023a), Indonesia’s rice production reached 53.63 million tonnes, a decrease of 2.05% compared to the previous year. This production is considered worrying compared to other countries when looking at the ratio to the population (Rozaki, 2020; Fitrawaty et al., 2023). This is crucial, considering Indonesia’s population growth rate is relatively high at 1.13% Statistic Indonesia (2023b). Therefore, innovations related to the sustainability of rice production must be further developed to maintain food security in Indonesia.

Other factors, such as the issue of climate change, also influence the stability and sustainability of rice production. Climate change is an environmental change due to increased greenhouse gas concentrations (Cassia et al., 2018; Karbi and Chemke, 2023; Simmer et al., 2023). The increase causes the trapping of reflected heat waves like the greenhouse system so that the earth’s temperature increases and has an impact on changing the rhythm of seasons and rainfall patterns in various parts of the world (Cassia et al., 2018; Marx et al., 2021; Karbi and Chemke, 2023). This can induce various plant abiotic stresses, such as drought, salinity, acidity, and other stresses (Sánchez-Bermúdez et al., 2022; Eckardt et al., 2023). However, water-related stress is the main problem due to climate change (Ahmed et al., 2020; Chen et al., 2023; Sato et al., 2024; Yadav et al., 2024). Water, as the main component of climate change, will increase the duration of dry and rainy season patterns, including their intensity, so that seasonal patterns are not more evident per year (Fahad et al., 2017; Ahmed et al., 2020; Haile et al., 2021; Chen et al., 2023; Janni et al., 2023; Yadav et al., 2024). In addition, these effects will also induce the intensity of the El Niño and La Niña effects to be more intense in several locations in the hemisphere (Yang et al., 2018; Geng et al., 2023; Wang et al., 2023), including Indonesia (Nur’utami and Hidayat, 2016; Arjasakusuma et al., 2018; Sulaiman et al., 2023). El Nino-Southern Oscillation (ENSO) is a climatic phenomenon that causes predominant rainfall variability in the tropics. The phenomenon causes severe droughts in many regions, causing many socio-economic losses (Rodysill et al., 2019; Adamson, 2022). On the other side, La Nina events cause an increase in convection and regional precipitation that drives heavy rainfall events. This causes major flooding in the region (Rodysill et al., 2019). Both phenomena effects cause a decrease in rice grain yield per hectare, leading to crop failure (Ansari et al., 2021; Barrios-Perez et al., 2021; Cherian et al., 2021; Haile et al., 2021). This is also the case in Indonesia, where the impact of El Nino and La Nina can reduce Indonesia’s rice production by around 2.9% to 4% (Murniati and Mutolib, 2020; Khairullah et al., 2021). In addition, according to (Sun et al., 2020), these two phenomena have an impact on reducing rice production, slowing down planting, and harvest failure, so they account for 40% of the variability in rice grain yield per hectare in Indonesia. Therefore, preventive measures need to be taken to adapt to the impacts of climate change. One innovation that can be offered is the development of early-maturing rice varieties.

The development of early maturing crop varieties is one of the adaptation strategies that aim to avoid the impact of climate stress (Rohaeni and Ishaq, 2016; Ren et al., 2023). This is in contrast to other adaptation strategies, such as avoidance and tolerance, which directly deal with climate stress (Seleiman et al., 2021; Eckardt et al., 2023; Yun et al., 2023; Anshori et al., 2024). In addition, this approach can optimize planting intensity under normal conditions so that rainfed land can be planted two to three times per year (Sudana, 2016; Subekti and Umar, 2022; Musa et al., 2023; Widiastuti et al., 2023; Sutardi et al., 2023). The development of early maturing varieties has also been conducted in rice and reported by Iftekharuddaula et al. (2016); Fang et al. (2019); Sjahril et al. (2020); Saminadane et al. (2023); Shanmugam et al. (2023). In general, the development of early maturing rice varieties in Indonesia has also been carried out, where there are several early maturing rice varieties that the Ministry of Agriculture has released (Purba and Giametri, 2017; Rismawati et al., 2022; Musa et al., 2023). These varieties have been adapted in several regions. However, specific reports related to interaction analysis, as one of the bases for evaluation, have yet to be published among these early maturing varieties. This includes comparisons of potential with high-yielding varieties in general. Nevertheless, Musa et al. (2023) and Anshori et al. (2024) have reported the potential fertilizer response to NPK dosage and its yield adaptability among five potential early maturing varieties. However, the concept has yet to cover the multivariate comprehensive influence of all growth characters on the stability and recovery responsiveness of the early maturing rice. In general, grain yield per hectare is not a dependent character. In other words, its variability is influenced by other agronomic components (Fellahi et al., 2018; van Eeuwijk et al., 2019; Anshori et al., 2021; Burgess et al., 2023; Hassani et al., 2023). This indicates that rice stability and responsiveness testing require integrated evaluation support criteria (Hashim et al., 2021; Salah et al., 2022; Santos et al., 2022; Hassani et al., 2023). The concept was also reported by (Alsabah et al., 2019; Akbar et al., 2021; Hashim et al., 2021; Salah et al., 2022). Therefore, several supporting characters must be integrated and synergistically included in the evaluation and interaction analysis.

Interaction analysis is an approach to measure a genotype’s response level to environmental changes. This concept is crucial for further evaluating a line or the recommendation process of a variety to be adapted to a region (Saltz et al., 2018; Smith et al., 2021; Gupta et al., 2022). These advantages make this analysis often applied in breeding activities, known as GxE interaction analysis (Yang, 2014; Brown et al., 2020; Gupta et al., 2022). Several concepts of GxE interaction analysis have been reported by several rice studies (Oladosu et al., 2017; Poli et al., 2018; Hashim et al., 2021; Roy et al., 2022; Panda et al., 2023; Ghazy et al., 2024). However, the specific assessment of GxE interactions in early-maturing rice has yet to be widely reported. In addition, analyses of GxE interactions are generally independent between growth characters and even only focused on grain yield per hectare (Yang, 2014; Brown et al., 2020; Hilmarsson et al., 2021; Roy et al., 2022). However, some studies have linked their potential using multivariate analyses (Sitaresmi et al., 2019; Pour-aboughadareh and Sanjani, 2021; Sumuni et al., 2024). Alam et al. (2021); Hashim et al. (2021); Hasan et al. (2022); Roy et al. (2022); Aswidinnoor et al. (2023); Singh et al. (2023), and Panda et al. (2023) used the principal component analysis pattern in analyzing the potential of a rice genotype to several environments. In addition, Sae-Lim et al. (2014); Mengesha et al. (2019), and Smith et al. (2021) also developed the concept of interaction analysis on a genotype through factor analysis. However, both multivariate analysis concepts still emphasize one main characteristic: grain yield per hectare. Meanwhile (Olivoto et al., 2019a, b), Sharifi et al. (2020); Pour-aboughadareh and Sanjani (2021); Panda et al. (2023), and Ahmed et al. (2024) also utilized the potential of various traits through the concept of the weighted average of absolute scores (WAASB), commercial check variety which utilized multivariate analysis and indices. However, these concepts only focus on stability, so the idea is considered less comprehensive in describing the potential for responsiveness between genotypes. Based on this, the development of a new approach that is more comprehensive in assessing the responsiveness of a GxE interaction needs to be done, especially for early maturing rice varieties. This study aims to develop and evaluate the effectiveness of multivariate approaches in analyzing GxE interactions that are more comprehensive and responsive. In addition, this study also aims to assess and determine the interaction potential of early maturing rice varieties that are responsive and have the potential to be developed.

## 2 Materials and methods

Two districts were chosen for this study: Soppeng Regency, at coordinates of 4°20’44.698 “S, 119°54’54.032 “E, and Bone District, West Sulawesi, Indonesia, at coordinates of 4°36’30.975 “S, 120°17’41.636 “E.” Based on South Sulawesi’s potential for rice production—particularly in the island’s eastern region—both sites were selected. The evaluation activities used two growth seasons: January–April 2022 (1^st^ season) and June–September 2022 (2^nd^ season). **Table 1** shows the rainfall trends for each season.

**Table 1:** The rainfall trends for the 2022 year in both locations.

Locations	Soil type	Rainfall pattern in 2022 year (mm)											
		Jan	Feb	March	April	May	June	July	Aug	Sep	Oct	Nov	Dec
Bone	Dusty Clay	79	269	227	113	370	455	241	231	297	296	80	241
Soppeng	Clay	123	159	56	157	168	199	106	131	116	307	101	230

### 2.1 Experimental design

This study used a randomized complete block design, with replications nested in two seasons and two locations (4 environments). Two commercial check varieties (Ciherang and Inpari 32) and five early maturing varieties (Cakrabuana, Padjajaran, Inpari 13; Inpari 19, and M70D) were grown three times in each environment. The combination of varieties, replications, and environment resulted in 84 experimental units. Based on research by (Barokah et al., 2021; Subekti and Umar, 2022; Musa et al., 2023 and Anshori et al., 2024), five early maturing rice varieties were chosen. In the meantime, the two varieties (Ciherang and Inpari 32) in South Sulawesi potential demand and seed requirements are taken into consideration when selecting check varieties (Sitaresmi et al., 2023; Qadir et al., 2024).

### 2.2 Research procedure

The research methodology started with plowing and tilling the land to create a muddy environment. After that, the field was organized with a plot system measuring 3.5 m x 3.5 m and 1 m between plots. Simultaneously, the seeds to be planted were pre-soaked for 24 hours. Then, the seeds are mixed into the nursery bed. Seedlings were reared until 15 days old, and then transferred to the field with a spacing of 20 cm x 20 cm, resulting in 416 plants per plot (Anshori et al., 2024).

Seedlings planted are maintained with various activities such as replanting, weeding, watering, fertilizing, and insect control. Weeding is done manually and chemically at 30 days after planting. Dead seedlings are replaced seven days later by replanting according to the variety. Weeds were removed mechanically, followed by herbicide application using a sprayer. Irrigation began six days after the first fertilization or 20 days after planting by adding water to the experimental field about 5 cm above the soil surface. After the second fertilization, irrigation was temporarily stopped to keep the soil moist and clay-like. Watering was resumed five days later, with the water level raised to about 10 cm above the soil surface during the primordial phase to prevent the formation of additional tillers. Fourteen days after transplanting, a fertilizer mixture of 200 kg ha^-1^ N: 100 kg ha^-1^ P_2_O_5_: 100 kg ha^-1^ K_2_O was applied, followed by a second round of urea fertilization 35 days later. Pest and disease control used pesticides adjusted to the type and phenological stage of the rice plant pest or disease. Harvesting is done when two-thirds of the rice panicles have reached physiological maturity (yellowing straw), and the grain at the base of the panicle has hardened. Data were collected throughout the harvesting process before the plant portions were placed into the sample bags.

### 2.3 Observation parameters and data analysis

This study focused on several agronomic characteristics of rice. These characters include plant height (PH), number of total tillers (NTT), number of productive tillers (NPT), days to harvesting (DH), flag leaf length (FLL), panicle length (PL), biological yield (BY), thousand-grain weight (TGW), and grain yield per hectare (GY). Biological yield means the average grain weight per hill from five sample plants, while grain yield is observed by converting plot weight to weight per hectare. These observational characters were systematically analyzed to evaluate the potential for GxE interactions, especially in early-maturing rice varieties. Meanwhile, the average of all growth traits to all locations and seasons was shown in **Supplementary Table 1**.

Data analysis began with a nested analysis of variance involving potential season and location interactions as nests in the replicates. The result of the analysis becomes the basis of whether further analysis is needed. If there are complex interactions on the observed characters, especially season x location x variety interactions, then GxE interaction analysis is conducted. The GxE interaction analysis starts with the best linear unbiased prediction (BLUP) analysis (Olivoto et al., 2019a, 2022; Schmidt et al., 2019; Khanna et al., 2022). Characters that did not meet the potential heritability of BLUP were not included in the multivariate analysis. Selected characters in BLUP were analyzed using factor analysis (Rocha et al., 2018; Olivoto et al., 2019a, 2022) and path analysis (Sabouri et al., 2008; Anshori et al., 2021; Fikri et al., 2023) as part of the multivariate analysis. The characters specified in the multivariate analysis were used as selection criteria in the index.

The index’s weighting is based on the combination of the three analyses: BLUP heritability, score on factor analysis, and direct effect of path analysis. The potential of each variety in the index is based on the standardized BLUP value. The standardized values are inputted into the index formulation of the previously developed multiple regression equation (Olivoto et al., 2019b; van Eeuwijk et al., 2019; Anshori et al., 2021, 2022).


Index=(0.315*0.974*0.48)DH_z+(0.464*0.792*0.54) BY_z+(0.404*0.720)GY_z

Or


Index=0.147 DH_z+0.199 BY_z+0.291 GY_z

Then, the index values were averaged and sorted per environment before regression analysis, like Finlay-Wilkinson’s stability testing, was conducted (Pour-Aboughadareh et al., 2022; Anshori et al., 2024). The interaction analysis results based on the index values were tested for sensitivity through the determination value (R^2^) and compared with the independent GY potential. This was to see the potential effectiveness between the two approaches.

## 3 Results

Variance analysis showed that the varietal diversity source significantly affected all growth characters (**Table 2**). In contrast, season and location-independent sources of diversity only significantly affected some characters, such as PH, NPT, BY, TGW, and grain yield per hectare (GY) influenced by season, and NTT, NPT, and BY influenced by location. Meanwhile, the interaction source of diversity only has a specific effect on several characters. By character, BY is a character significantly affected by all sources of diversity. This is followed by the number of productive tiller characters, which is also influenced by all sources of diversity, except for the season x location x genotype interaction. In contrast, the characters are influenced by a few sources of diversity, such as FLL and PL. Both characters are only influenced by the source of varietal diversity.

**Table 2:** Analysis of variance (ANOVA) of the interaction of season, environment, and genotype.

Source of variation	Pr>F								
	PH	NTT	NPT	DH	FLL	PL	BY	TGW	GY
Season	<.0001^**^	0.3123	0.0002^**^	0.2339	0.2996	0.0612	<.0001^**^	0.0005**	0.0002^**^
Location (loc)	0.4478	0.0310^*^	<.0001^**^	0.0014^**^	0.8223	0.1419	<.0001^**^	0.7633	0.3093
Season x loc	0.0051^**^	<.0001^**^	<.0001^**^	0.0786	0.1439	0.549	0.004^**^	0.707	0.7817
Replication/season x loc	0.9823	0.9174	0.0048^**^	0.8351	0.9979	0.9091	0.0455^*^	0.9778	0.6401
Variety (var)	<.0001^**^	<.0001^**^	0.0064^**^	<.0001^**^	<.0001^**^	<.0001^**^	<.0001^**^	<.0001^**^	<.0001^**^
Season x var	0.0013^**^	0.2038	<.0001^**^	0.0007^**^	0.9198	0.6311	<.0001^**^	0.9286	0.3872
Loc x var	0.3458	0.0260^*^	0.0094^**^	0.1236	0.5738	0.1492	0.0081^**^	<.0001^**^	0.0651
Season x loc x var	0.2095	0.109	0.5167	0.9265	0.959	0.451	0.0074^**^	0.4872	0.299

DF, degrees of freedom; CV, coefficient of variance; p-value, the probability under the assumption of no effect or no difference (null hypothesis), 0.05>p value > 0.01 is significant at 5% level (^*^), 0.01>p value is significant 1% level (^**^), PH, plant height; NTT, number of total tillers; NPT, number of productive tillers; DH, days to harvesting; FLL, flag leaf length; PL, panicle length; BY, biological yield; TGW, thousand-grain weight; GY, Grain yield per hectare.

The results of the BLUP analysis show that three characters have the same average BLUP value in each variety, namely NTT, NPT, and TGW (**Table 3**). This is followed by a heritability value that shows 0. Conversely, other characters have a variance of BLUP values between each variety, followed by heritability values above 0. PH (44.19%) and PL (23.5%) are characters with moderate heritability. Meanwhile, characters classified as high heritability are DH (97.35%), BY (79.24%), and GY (71.96%). Varieties with the highest BLUP GY values were Inpari 32 (8.54 tonnes/ha) and Ciherang (8.44 tonnes/ha) as a commercial check variety and Inpari 13 (7.83 tonnes/ha) as an early maturing rice variety.

**Table 3:** BLUP analysis of GXE interaction on rice growth characters.

Genotype	PH (cm)	NTT	NPT	DH (days)	FLL (cm)	PL (cm)	By (g)	TGW (g)	GY (ton/ha)
Cakrabuana	104.83	28.4	24.1	95.52	34.47	25.26	67.53	27.45	7.37
Ciherang	104.44	28.4	24.1	108.26	34.67	25.23	85.82	27.45	8.44
Inpari 13	107.19	28.4	24.1	98.12	35.24	25.38	76.26	27.45	7.83
Inpari 19	107.26	28.4	24.1	91.55	35.31	25.54	67.19	27.45	6.91
Inpari 32	101.87	28.4	24.1	116.29	34.47	24.87	82.49	27.45	8.54
M70D	105.98	28.4	24.1	88.95	35.12	25.01	66.11	27.45	6.83
Padjajaran	104.36	28.4	24.1	97.23	35.33	25.46	68.91	27.45	7.29
Heritability (%)	44.19	0.00	0.00	97.35	18.7	23.5	79.24	0.00	71.96

PH, plant height; NTT, number of total tillers; NPT, number of productive tillers; DH, days to harvesting; FLL, flag leaf length; PL, panicle length; BY, biological yield; TGW, thousand-grain weight of 1000 grains; GY, grain yield per hectare.

The factor analysis results showed that two dimensions could describe the representative diversity of the diverse characters based on the BLUP value (**Table 4**). In general, the commonality of all characters has reached 0.8, except for FLL, which only reached 0.769. Based on GY characters, factor 1 is the factor that collects the highest loading factor score for GY characters. In factor 1, the characters PH (0.052) and FLL (0.104) are the characters that have low loading scores. The DH, PL, and BY characters in factor 1 had good factor loading scores of 0.315, 0.271, and 0.464, respectively. Meanwhile, the results of the path analysis are shown in **Table 5**, which has a determination value of 0.84. DH and BY are two characters with a direct effect of 0.48 and 0.54, respectively. In contrast, PL had a low direct effect of 0.02 and was ineffective as a selection criterion.

**Table 4:** The Factor Analysis of BLUP values.

Variable	Factor 1	Factor 2	Communality
PH	0.052	-0.361	0.802
DH	0.315	-0.026	0.966
FLL	0.104	-0.406	0.769
PL	0.271	-0.561	0.815
BY	0.464	-0.238	0.953
GY	0.404	-0.143	0.987
Variance (Var)	2.9039	2.3872	5.2911
% Var	0.484	0.398	0.882

PH, plant height; DH, days to harvesting; FLL, flag leaf length; PL, panicle length; BY, biological yield; grain yield per hectare.

**Table 5:** Path analysis of selected characters on grain yield per hectare.

Character	Direct effect	Indirect effect			Residual
		DH	PL	BY	
DH	0.48		-0.01	0.48	0.03
PL	0.02	-0.24		-0.21	0.03
BY	0.54	0.42	-0.01		0.03

DH, days to harvesting; PL, panicle length; BY, biological yield.

The selection index combines standardized BLUP values with the weighting values developed in this study (**Table 6**). Based on the index value, the Bone_season 1 (E1) environment (1.71) is the environment with the highest average index, followed by Soppeng_season 1 (E3) with a value of 0.76. Inpari 32 (3.94 and 3.01, respectively) and Ciherang (2.93 and 1.96) were the best varieties in the E1 and E3 environments. In addition, Inpari 13 was also rated as the best variety, especially against other early-maturing varieties (1.52 and 1.40, respectively). On the contrary, the lowest average index value belongs to Environment_Soppeng season 2 with a value of (-2.01). Inpari 32, Ciherang, and Inpari 13 had index values of 0.6 -0.44 and -2.18, respectively. In contrast, the M70D variety has the lowest value in almost all environments, especially in the Soppeng season 2 environment.

**Table 6:** Selection index based on BLUP value of selected characters in each environment.

Environment	Genotype	BLUP Value			z standardization			Index
		DH	BY	GY	DH_z	BY_z	GY_z	
1	Cakrabuana	97.34	95.43	8.37	-1.29	4.73	1.52	1.19
1	Ciherang	104.98	101.32	9.74	3.46	6.00	4.22	2.93
1	Inpari 13	99.34	95.71	8.60	-0.05	4.79	1.98	1.52
1	Inpari 19	92.69	92.44	8.22	-4.18	4.08	1.23	0.56
1	Inpari 32	118.27	99.08	9.55	11.71	5.51	3.86	3.94
1	M70D	90.70	92.58	8.91	-5.41	4.11	2.58	0.77
1	Padjajaran	101.00	86.73	8.21	0.98	2.85	1.20	1.06
E1 (Bone_Season 1) Mean		100.62	94.75	8.80	0.75	4.58	2.37	1.71
2	Cakrabuana	97.01	56.49	7.07	-1.50	-3.66	-1.05	-1.25
2	Ciherang	116.64	84.46	9.90	10.70	2.37	4.54	3.37
2	Inpari 13	98.67	60.29	8.11	-0.46	-2.84	1.01	-0.34
2	Inpari 19	93.35	46.20	5.14	-3.77	-5.87	-4.86	-3.14
2	Inpari 32	112.65	70.78	10.77	8.22	-0.58	6.25	2.91
2	M70D	90.68	46.42	5.14	-5.43	-5.82	-4.86	-3.37
2	Padjajaran	97.34	59.13	6.55	-1.29	-3.09	-2.07	-1.41
E2 (Bone_Season 2) Mean		100.90	60.54	7.53	0.92	-2.78	-0.15	-0.46
3	Cakrabuana	90.23	85.86	7.49	-5.71	2.67	-0.22	-0.37
3	Ciherang	100.13	100.75	8.86	0.44	5.87	2.49	1.96
3	Inpari 13	95.02	106.09	8.30	-2.73	7.02	1.37	1.40
3	Inpari 19	87.35	93.40	7.73	-7.50	4.29	0.25	-0.18
3	Inpari 32	118.35	95.20	8.37	11.76	4.68	1.51	3.10
3	M70D	83.52	87.70	7.21	-9.88	3.06	-0.76	-1.06
3	Padjajaran	93.74	93.95	7.84	-3.53	4.41	0.47	0.49
E3 (Soppeng_Season 1) Mean		95.48	94.70	7.97	-2.45	4.57	0.73	0.76
4	Cakrabuana	97.34	35.89	6.31	-1.29	-8.09	-2.54	-2.54
4	Ciherang	111.99	56.69	6.09	7.81	-3.61	-2.98	-0.44
4	Inpari 13	99.33	38.04	6.47	-0.05	-7.63	-2.24	-2.18
4	Inpari 19	92.34	35.84	5.81	-4.40	-8.10	-3.54	-3.29
4	Inpari 32	116.65	65.80	6.47	10.71	-1.65	-2.23	0.60
4	M70D	90.34	39.39	5.42	-5.64	-7.34	-4.29	-3.54
4	Padjajaran	96.67	35.61	6.16	-1.71	-8.15	-2.85	-2.70
E4 (Soppeng_Season 2) Mean		100.67	43.90	6.10	0.78	-6.37	-2.95	-2.01

E, environment; DH, days to harvesting; BY, biological yield; GY, grain yield per hectare; _z, standardized value.

**Figure 1:**
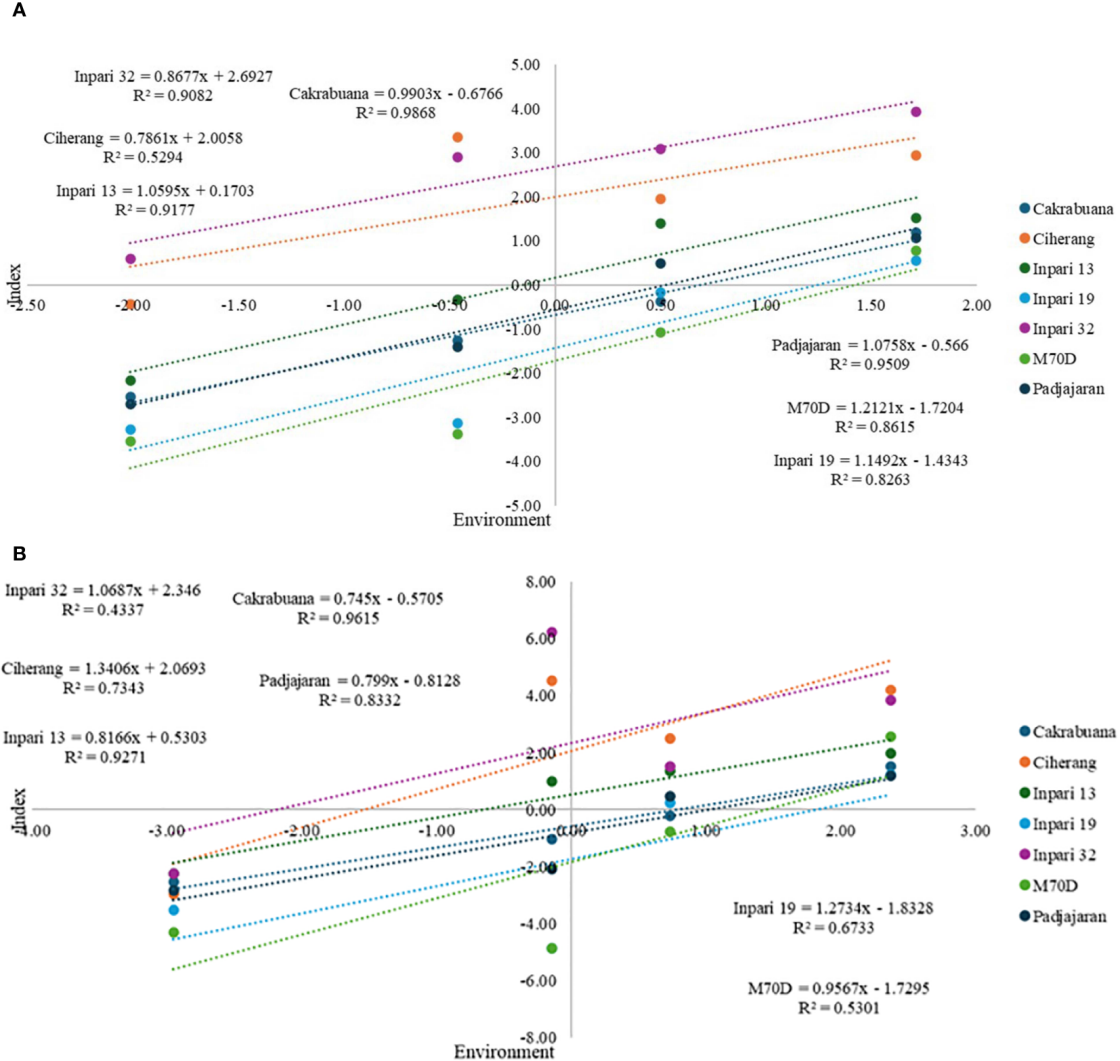
GxE interaction analysis is based on a multivariate index of selection criteria **(A)**, and GxE interaction analysis is based on grain yield per hectare **(B)**.

Based on the regression analysis results (**Figures 1A, B**), the coefficient of determination (R^2^) in the multivariate index-based GxE analysis has a high value above 0.8, except for the Ciherang variety. In contrast, the coefficient of determination in GY-based GxE analysis is relatively below 0.8, except for Inpari 13 (0.927), Cakrabuana (0.9615), and Padjajaran (0.8332). Ciherang variety in index-based GxE analysis has the lowest determination value, 0.529, with a regression gradient (b) of 0.786. The gradient is also relatively in the same group as the regression gradient of Inpari 32 (0.868). Regression gradients that have response values ranging from 1 ± 0.1 are Inpari 13 (1.06), Cakrabuana (1.99) and Padjajaran (1.07). In contrast, based on GxE GY, Inpari 32 and Inpari 19 have gradients ranging from 1 ± 0.1. However, both have low determination values of 0.4337 and 0.6733, respectively. Meanwhile, Inpari 13, Cakrabuana, and Padjajaran have regression gradients below 0.9.

## 4 Discussions

The ANOVA results showed that the sources of diversity in this study had specific and diverse patterns of influence among characters. However, one source of variation (variety) significantly affected all growth characters. Significant varietal diversity is due to differences in the types of varieties used, where there are five early maturing rice varieties and two medium maturing varieties as commercial varieties in general. The difference in type will correlate with phenology and agronomic characters (Paiman et al., 2022; Sheng et al., 2022; Afa et al., 2023; Seck et al., 2023), so the difference in growth response will be significant for all characters. As for the effect of interactions, the diversity of interactions is specific depending on the character and interaction pattern. However, in the character of biological yield, all sources of diversity showed a significant effect on the character. In general, biological yield is one of the key characteristics that can represent the potential for GY (Bhati et al., 2015; Li et al., 2019). This is also in line with Li et al. (2019) and Burgess et al. (2023), where biological yield is the accumulation of a potential GY component, so this character is often used as the main supporting criteria for the crop yield (Bhati et al., 2015; Li et al., 2019; Zhao et al., 2020; Dhakal et al., 2021). Based on this, this character can be a consideration in in-depth analysis, especially for more comprehensive interactions. However, in this study, GY was only influenced by independent variance sources of variety and season. Therefore, BLUP and multivariate analysis can be used in this study.

The results of estimating the mean value of BLUP-based characters explain that several characters (NTT, NPT, and TGW) do not have diversity between each genotype. In general, the estimation of the BLUP value is influenced by the potential genetic variation (Molenaar et al., 2018; Schmidt et al., 2019; Khanna et al., 2022). Suppose a character has a low genetic variance. In that case, it will impact the narrow variance and does not have a random effect on the BLUP value. So, the heritability of the three characters is 0 in the BLUP analysis (Molenaar et al., 2018; Sood et al., 2020). However, when comparing these results with the ANOVA, there needs to be more alignment because the varietal diversity source significantly affects all three characters. This indicates good heritability potential in the three characters (Fadhilah et al., 2022; Anshori et al., 2024). The difference is due to the BLUP concept, which is that the combination of season and location is combined into an environment. This will impact the pattern of diversity effects (Johnstone and Manly, 2014), so the heritability of the BLUP pattern will be lower than that of the ANOVA pattern in this study. In addition, according to Jia (2017), the difference in heritability between regular and BLUP has a different approach, where the BLUP heritability value that links cross-validation tends to be lower than the regular heritability value. Based on these two things, the potential in the ANOVA results will be different from the BLUP potential. However, the BLUP potential value is believed to be more effective in assessing the potential of a genotype than the general approach. This is because the BLUP approach can correct the potential value of the genotype to the influence of its environment so that the potential assessment is more accurate, especially for multi-environment experiments (MET) (Molenaar et al., 2018; Schmidt et al., 2019; Sood et al., 2020; Chen et al., 2024). Therefore, NTT, NPT, and TGW characters were not included in subsequent analyses.

The combination of several multivariate analyses is a systematic approach to determining selection or evaluation criteria (Kose et al., 2018; Hashim et al., 2021; Laraswati et al., 2021; Barth et al., 2022; Fadhilah et al., 2022; Habib et al., 2024). These criteria can increase the effectiveness of the selection process. In this study, the combination of multivariate analyses focused on factor analysis and cross-sectional analysis. Both analyses were chosen, given their potential to reduce the diversity of ineffective characters in evaluation judgments (Nayak et al., 2018; Anshori et al., 2022; Farid et al., 2022; Fikri et al., 2023). Generally, factor analysis can reduce diversity in characters with low covariance in a dimension. This makes the character unimportant in influencing the variety of dimensions so that the character can be eliminated in the evaluation process (Nayak et al., 2018; Rocha et al., 2018; Tavakol and Wetzel, 2020; Jia et al., 2022). This concept is also suggested when analyzing the interaction and stability of a genotype. Rocha et al. (2018) and Olivoto et al. (2022) Utilized BLUP values and factor analysis to assess genotype potential. However, the concept still needs to be approached with path analysis that focuses on the direct effect of a character on the total diversity of a main character (Anshori et al., 2022; Fikri et al., 2023). This is due to the importance of a unidirectional evaluation of the main characters that can describe and answer the objectives of an assessment (Fikri et al., 2023; Musa et al., 2023). Therefore, combining factor and path analysis as part of multivariate analysis is considered effective in estimating selection criteria in evaluating GxE interactions.

GxE interaction analyses of DH, BY, and GY can be done independently. The concept of independent assessment of selection or evaluation criteria in rice was also reported by Hilmarsson et al. (2021) and Musa et al. (2023). However, the three characters are related, so the assessment should combine them. One of them is through the index value approach. The utilization of index values in the combination of evaluation and selection criteria has also been reported by (Fadhilah et al., 2022; Sarwendah et al., 2022; Fikri et al., 2023). In general, the index value becomes the midpoint in combining the advantages and disadvantages of a genotype against various selection or evaluation criteria (Olivoto et al., 2019b; van Eeuwijk et al., 2019; Fikri et al., 2023). The combination involves dimensional adjustment so that the three criteria can be combined in a linear equation with balanced values (Sabouri et al., 2008; Anshori et al., 2021, 2022; Batista et al., 2021; Fikri et al., 2023). This assesses the interaction of the three comprehensive and objective (Sabouri et al., 2008; Anshori et al., 2021; Batista et al., 2021). The utilization of index values based on BLUP analysis was also reported by Olivoto et al. (2019b); Pour-aboughadareh and Sanjani (2021), and Panda et al. (2023). However, the combination of the three also considers the priority level of a criterion, so weighting in the index is necessary (Sabouri et al., 2008; van Eeuwijk et al., 2019; Pavithra and Vengadessan, 2020; Anshori et al., 2021; Batista et al., 2021; Rahimi and Debnath, 2023). Consideration of the weighting value can use the heritability approach (Fadhilah et al., 2022; Rahimi and Debnath, 2023; Farid et al., 2024), factor score in factor analysis (Anshori et al., 2022; Farid et al., 2022; Fikri et al., 2023), or direct effect on cross-section (Sabouri et al., 2008; Alsabah et al., 2019; Pavithra and Vengadessan, 2020; Anshori et al., 2021, 2022; Sarwendah et al., 2022; Fikri et al., 2023). The three concepts are considered effective in assessing selected and evaluated genotypes, so combining the three can be a new approach to forming index values for GxE analysis. This is also supported by reports on the effectiveness of combining various genetic and multivariate analyses in forming selection indices (Farid et al., 2022; Fadhilah et al., 2022).

The GxE interaction analysis in this study was combined with the orthogonal-polynomial concept based on regression analysis of several varieties. This concept is very familiar in stability analysis by Finlay-Wilkinson, which focused on simple linear regression (Finlay and Wilkinson, 1963). In addition, the development of regression-based GxE interaction analysis was also reported by Brown et al. (2020); Hilmarsson et al. (2021) and Roy et al., 2022). However, the approach in this study focuses on the index value of the combination of the three selection criteria, so the regression analysis focuses on the concept of multiple regression. This makes the interaction assessment simpler, more structured, and more comprehensive, making the results easy to understand and interpret in the evaluation process.

Based on the interaction index analysis approach, all rice varieties have high determination values reaching > 0.8, except for the Ciherang variety (0.529). In general, the determination value above 0.8 indicates a high level of effectiveness in a model. This is inversely proportional to GY-based GxE analysis, which has a determination value below 0.8. This suggests that the index approach effectively assesses multivariate rice growth interaction responses, especially for early maturing varieties. In addition, in this analysis, Inpari 13, Cakrabuana, and Padjajaran varieties have a stable response to environmental changes. This is characterized by b values close to or equal to 1, like the Finlay-Wilkinson concept (Finlay and Wilkinson, 1963; Piepho and Blancon, 2023; Anshori et al., 2024). In contrast, interactions based solely on GY had b values below 0.9 for all three varieties. These results indicate that the index approach can comprehensively assess rice growth characters’ responsiveness, so the three varieties are considered suitable for planting in various seasons at both test sites. This developed concept is different from the Finlay-Wilkinson interaction analysis and MGIDI, which can only assess partially whether only focusing on the potential responsiveness (Finlay-Wilkinson) or the comprehensive potential assessment (MGIDI) (Sitaresmi et al., 2019; Olivoto et al., 2022; Pour-Aboughadareh et al., 2022; Debsharma et al., 2023). This makes this approach the meeting point of the two interaction analyses, so the picture of responsiveness related to potential GxE interactions can be assessed comprehensively and systematically. Therefore, the orthogonal-polynomial multiple regression approach based on multivariate analysis and BLUP index values can be recommended for analyzing GxE interactions. In addition, Inpari 13, Cakrabuana, and Padjajaran rice varieties are recommended as adaptive varieties, especially in both locations (Bone and Soppeng).

## 5 Conclusions

In conclusion, the new approach through BLUP-based multiple regression index, factor analysis, and path analysis is considered adequate in analyzing GxE interactions, especially in evaluating early maturing rice. In addition, the approach is also considered to combine the concepts of Finlay-Wilconson and MGIDI stability analysis in the analysis of GxE interactions. Based on this approach, the index formed is 0.147*days to harvesting standardized + 0.199*biological yield standardized +

0.291*grain yield per hectare (GY) standardized. The index approach showed a high determination above 0.8 with a gradient (b) value above 0.9 in the GxE interaction analysis, especially for early maturing rice varieties. This compares favorably with GY-based GxE interaction analysis. Therefore, this index approach can be recommended in GxE interaction analysis, especially in evaluating early maturing rice genotypes. In addition, based on the index-based GxE interaction analysis, the early maturing rice varieties Inpari 13, Cakrabuana, and Padjajaran are recommended to be used as adaptive varieties, especially in both locations (Bone and Soppeng).

### 5.1 Resource identification initiative

The project uses STAR 2.0.1 from IRRI, META-R from CIMMYT, and the Microsoft Excel 2016 version. Seeds of rice varieties were obtained from rice seed markets in Indonesia.

## Data Availability

The original contributions presented in the study are included in the article/**Supplementary Material**. Further inquiries can be directed to the corresponding author.
