# Supplementary material for: A comprehensive multivariate approach for GxE interaction analysis in early maturing rice varieties
Source: Front Plant Sci. 2024 Oct 1;15:1462981. doi: 10.3389/fpls.2024.1462981 (PMC11473407; doi:10.3389/fpls.2024.1462981)
Supplement: Supplementary file 1 [file Table1.docx]

**Supplementary 1.** The average of growth traits to all varieties in each location and season

| Location | Season | Variety | PH (cm) | NTT | NPT | DH (days) | FLL (cm) | PL (cm) | BY (g) | TWG (g) | GY (ton ha-1) |
| --- | --- | --- | --- | --- | --- | --- | --- | --- | --- | --- | --- |
| Bone | 1 | Cakrabuana | 108.33 | 26.89 | 26.89 | 97.33 | 31.89 | 25.56 | 95.56 | 27.78 | 8.37 |
| Bone | 1 | Ciherang | 105.78 | 27.17 | 25.67 | 105.00 | 34.66 | 27.64 | 102.56 | 30.72 | 9.74 |
| Bone | 1 | Inpari 13 | 103.67 | 25.61 | 25.61 | 99.33 | 33.73 | 25.59 | 95.89 | 28.78 | 8.60 |
| Bone | 1 | Inpari 19 | 110.39 | 26.28 | 26.28 | 92.67 | 36.79 | 27.20 | 92.00 | 28.67 | 8.22 |
| Bone | 1 | Inpari 32 | 102.83 | 26.78 | 26.78 | 118.33 | 32.63 | 24.43 | 99.89 | 30.44 | 9.56 |
| Bone | 1 | M70D | 105.55 | 26.33 | 26.33 | 90.67 | 44.10 | 24.75 | 92.17 | 28.61 | 8.91 |
| Bone | 1 | Padjajaran | 108.00 | 26.06 | 26.06 | 101.00 | 43.24 | 26.13 | 85.22 | 27.33 | 8.21 |
| Mean | | | 106.36 | 26.44 | 26.23 | 100.62 | 36.72 | 25.90 | 94.75 | 28.90 | 8.80 |
| Bone | 2 | Cakrabuana | 94.56 | 37.00 | 27.45 | 97.00 | 27.44 | 21.59 | 55.91 | 25.50 | 7.04 |
| Bone | 2 | Ciherang | 98.11 | 44.00 | 36.33 | 116.67 | 29.22 | 24.48 | 87.90 | 26.23 | 10.08 |
| Bone | 2 | Inpari 13 | 104.67 | 44.45 | 30.00 | 98.67 | 33.00 | 24.05 | 60.26 | 24.17 | 8.15 |
| Bone | 2 | Inpari 19 | 101.45 | 38.22 | 33.22 | 93.33 | 30.11 | 22.38 | 44.14 | 22.50 | 4.96 |
| Bone | 2 | Inpari 32 | 100.22 | 36.78 | 29.44 | 112.67 | 31.89 | 24.41 | 72.25 | 25.60 | 11.01 |
| Bone | 2 | M70D | 106.00 | 31.78 | 25.22 | 90.67 | 33.55 | 23.47 | 44.39 | 22.90 | 4.96 |
| Bone | 2 | Padjajaran | 104.33 | 35.55 | 28.45 | 97.33 | 35.78 | 24.82 | 58.93 | 28.07 | 6.48 |
| Mean | | | 101.33 | 38.25 | 30.02 | 100.90 | 31.57 | 23.60 | 60.54 | 25.00 | 7.53 |
| Soppeng | 1 | Cakrabuana | 118.93 | 28.27 | 26.00 | 90.00 | 36.67 | 28.56 | 78.67 | 29.79 | 7.34 |
| Soppeng | 1 | Ciherang | 111.20 | 30.47 | 27.87 | 100.33 | 33.89 | 23.22 | 105.67 | 23.94 | 9.14 |
| Soppeng | 1 | Inpari 13 | 120.80 | 30.07 | 28.13 | 95.00 | 39.11 | 27.33 | 115.33 | 29.88 | 8.40 |
| Soppeng | 1 | Inpari 19 | 124.13 | 25.20 | 22.80 | 87.00 | 42.33 | 28.34 | 92.33 | 28.22 | 7.65 |
| Soppeng | 1 | Inpari 32 | 90.13 | 25.47 | 22.87 | 119.33 | 28.11 | 22.00 | 95.60 | 30.65 | 8.49 |
| Soppeng | 1 | M70D | 115.53 | 38.20 | 35.33 | 83.00 | 33.00 | 24.89 | 82.00 | 29.16 | 6.98 |
| Soppeng | 1 | Padjajaran | 112.87 | 31.07 | 28.40 | 93.67 | 33.22 | 29.67 | 93.33 | 27.48 | 7.80 |
| Mean | | | 113.37 | 29.82 | 27.34 | 95.48 | 35.19 | 26.29 | 94.70 | 28.45 | 7.97 |
| Soppeng | 2 | Cakrabuana | 96.00 | 16.00 | 13.00 | 97.33 | 33.55 | 25.44 | 33.74 | 28.95 | 6.36 |
| Soppeng | 2 | Ciherang | 99.22 | 24.45 | 20.00 | 112.00 | 36.11 | 25.34 | 60.12 | 27.32 | 6.09 |
| Soppeng | 2 | Inpari 13 | 110.00 | 16.78 | 12.33 | 99.33 | 40.33 | 26.22 | 36.47 | 26.35 | 6.55 |
| Soppeng | 2 | Inpari 19 | 103.78 | 15.78 | 12.67 | 92.33 | 38.45 | 28.00 | 33.68 | 25.14 | 5.74 |
| Soppeng | 2 | Inpari 32 | 97.78 | 21.22 | 18.11 | 116.67 | 36.95 | 23.67 | 71.68 | 30.72 | 6.55 |
| Soppeng | 2 | M70D | 101.11 | 19.55 | 15.11 | 90.33 | 32.89 | 23.78 | 38.19 | 25.97 | 5.27 |
| Soppeng | 2 | Padjajaran | 88.34 | 19.11 | 13.56 | 96.67 | 35.78 | 24.00 | 33.39 | 25.22 | 6.17 |
| Mean | | | 99.46 | 18.98 | 14.97 | 100.67 | 36.29 | 25.21 | 43.90 | 27.10 | 6.10 |
| Mean overall | | | 105.13 | 28.38 | 24.64 | 99.42 | 34.94 | 25.25 | 73.47 | 27.36 | 7.60 |

Note : PH: plant height, NTT: number of total tillers, NPT: number of productive tillers, DH: days to harvesting, FLL: flag leaf length, PL: panicle length, BY: biology yield, TGW: thousand-grain weight, GY: Grain yield per hectare
